# Considerations for resting state functional MRI and functional connectivity studies in rodents

**DOI:** 10.3389/fnins.2015.00269

**Published:** 2015-08-05

**Authors:** Wen-Ju Pan, Jacob C. W. Billings, Joshua K. Grooms, Sadia Shakil, Shella D. Keilholz

**Affiliations:** ^1^Department of Biomedical Engineering, Georgia Institute of Technology, Emory UniversityAtlanta, GA, USA; ^2^Neuroscience Program, Emory UniversityAtlanta, GA, USA; ^3^School of Electrical and Computer Engineering, Georgia Institute of TechnologyAtlanta, GA, USA

**Keywords:** resting state fMRI, resting state functional connectivity, rodent models, anesthesia, preclinical models

## Abstract

Resting state functional MRI (rs-fMRI) and functional connectivity mapping have become widely used tools in the human neuroimaging community and their use is rapidly spreading into the realm of rodent research as well. One of the many attractive features of rs-fMRI is that it is readily translatable from humans to animals and back again. Changes in functional connectivity observed in human studies can be followed by more invasive animal experiments to determine the neurophysiological basis for the alterations, while exploratory work in animal models can identify possible biomarkers for further investigation in human studies. These types of interwoven human and animal experiments have a potentially large impact on neuroscience and clinical practice. However, impediments exist to the optimal application of rs-fMRI in small animals, some similar to those encountered in humans and some quite different. In this review we identify the most prominent of these barriers, discuss differences between rs-fMRI in rodents and in humans, highlight best practices for animal studies, and review selected applications of rs-fMRI in rodents. Our goal is to facilitate the integration of human and animal work to the benefit of both fields.

## Introduction

Resting state MRI (rs-fMRI) and functional connectivity measurements have become extensively used tools in the human neuroimaging community, mapping networks of coordinated activity within the brain. Functional connectivity measurements have provided insight into normal brain function, development, neurological disorders, aging, and psychiatric conditions (Lowe et al., 2002; Greicius et al., 2004; Fox et al., 2007; Calhoun et al., 2008; Kennedy and Courchesne, 2008; Thompson et al., 2013a). Because there is no need for a task, rs-fMRI facilitates the study of brain function among populations where task-based functional MRI (fMRI) can be challenging.

**Figure 1:**
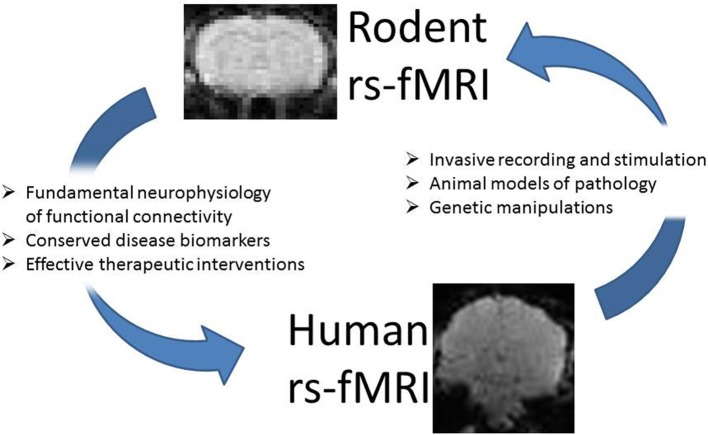
**Resting state MRI studies in rodents and humans are closely interwoven**. Work in rodent models can provide insight into the neurophysiology underlying functional connectivity and the alterations in connectivity observed in different disorders. The application of the technique in animal models can identify potential biomarkers and suggest targets for intervention in humans. Similarly, findings in the human population can suggest targets for multimodal studies that provide new insight into observed alterations. Functional connectivity may be used as a biomarker to evaluate how well an animal model reproduces the deficits observed in humans, or in conjunction with genetic manipulations to understand the origins of network alterations.

Biswal et al. first reported resting state functional connectivity in human subjects in 1995 (Biswal et al., 1995), but the first demonstrations of resting state connectivity in an animal model came a decade later, when the growing application of the technique in humans made the need for a better understanding of its neurophysiological basis apparent (Lu et al., 2006, 2007; Williams et al., 2006, 2010; Pawela et al., 2008; Zhao et al., 2008). Following the success of animal models in characterizing the blood oxygenation level dependent (BOLD) response to stimulation, these studies began to address the similar problem of understanding the sources of the spontaneous BOLD fluctuations used to map functional connectivity (Lu et al., 2007; Shmuel and Leopold, 2008; Magnuson et al., 2010; Schölvinck et al., 2010; Pan et al., 2011, 2013; Liu et al., 2013b). As the application of rs-fMRI in human studies grows, the technique is increasingly being translated to the examination of animal models of neurological and psychiatric disorders. Beginning with a handful of articles describing functional connectivity and its physiological basis in the rat in 2007–2008, the literature has grown to 45 articles involving rs-fMRI in mice and rats in 2014 alone, which examine the effects of depression, drug abuse, plasticity, prenatal stress, and more (Alvarez-Salvado et al., 2014; Goelman et al., 2014; Lu et al., 2014; Williams et al., 2014). Today, research seeking to understand functional connectivity is divided between studies performed on human subjects and studies performed on animal models. While work in humans continues to demonstrate how functional brain networks contribute to physiology and behavior in a way that is immediately relevant to our species, animal research presents an opportunity to examine these relationships at a level that is otherwise impermissible. In this way, the two are intricately interwoven, and it is often the case that recent findings from one species drive current research in the other. The observation of behaviorally and functionally relevant alterations in functional networks in humans motivates animal studies that examine the neurophysiological basis of the alterations, which in turn allows human studies that use the technique to gain insight into pathological and normal brain function. One of the major strengths of functional connectivity mapping is its role as a translational tool that can investigate disease models or scientific hypotheses in a well-controlled environment (Figure 1).

The field of neuroimaging clearly benefits from the interaction between human and animal investigations, but there are barriers that impede simple translation from human studies to animal work and back again. In human studies, for example, it is usually taken for granted that the subject is in approximately the same physiological condition for the entire rsfMRI experiment (unless they fall asleep). In animal rs-fMRI, maintaining stable physiology within a normal range is typically more complicated due to the use of anesthesia but remains a crucial step in order to obtain reliable, interpretable data. Many non-neural sources of physiological fluctuations can result in hemodynamic changes that may equal or even surpass the amplitude of neurally-related BOLD changes. While anesthesia alleviates stress and prevents motion, it also affects neurovascular coupling, another complication that must be considered when interpreting findings. Furthermore, while the general data preprocessing pipeline is similar for humans and rodents, some steps are relatively more important for data quality control in rodents, and specific strategies for data analysis must be adapted from human rs-fMRI. In this review we identify some of the barriers to translation across species, discuss differences between rs-fMRI in animals and in humans, highlight best practices for animal studies, and review selected rodent rs-fMRI studies as examples of the power of the technique. Our goal is to facilitate the integration of human and animal work to the benefit of both fields.

### Comparability across species

Most functional connectivity studies in animals to date have been performed in non-human primates or rodents (Lu et al., 2007; Pawela et al., 2008; Shmuel and Leopold, 2008; Zhao et al., 2008; Williams et al., 2010; Hutchison et al., 2013b). Studies in non-human primates are valuable because their brains are relatively similar to humans but invasive techniques can provide data not obtainable from healthy human subjects (Hutchison and Everling, 2012). However, work in non-human primates is difficult and expensive, limiting their utility in large-scale translational research. Furthermore, the considerations for rs-fMRI are often more similar to those for humans than to those required for rodents. For this reason, we will limit this review to rodent models.

Understanding the close homology between human and rodent brains offers a perspective on why comparative neuroscience studies in rodents have proven to be so useful. Both belong to the subclass of placental mammals who flourished in the years following the ecological upheaval demarcated by K/Pg boundary of the geological timeline, ~65 (Luo, 2007; O'Leary et al., 2013). Rostral to the brainstem and cerebellum common to all vertebrates, mammals share a common telencephalic organization consisting of basal ganglia, allocortex, and neocortex (Butts et al., 2014). In addition, placentalia share the common adaptation of a large inter-cortical commissure, the corpus callosum (Heath and Jones, 1971; Katz et al., 1983). Speciation within placentalia largely involves incremental changes to the neocortex, including changes in the size and configuration of cortical fields, in the number of cortical fields, and in the pattern of connections among homologous fields (Krubitzer, 1995). Common to all mammalian neocortices are primary and secondary representations of auditory, visual, and somatic sensory fields, multimodal association areas between these fields, and motor output regions (Kaas, 2007; Krubitzer, 2007). From work with stimulus-related functional MRI (fMRI), it is known that neurovascular coupling is relatively conserved across species, although specific features like the hemodynamic delay can differ (de Zwart et al., 2005). Further evidence that rs-fMRI arises from similar origins in rodents and humans comes from a number of studies showing that both relatively simple networks (somatosensory, motor, visual, and subcortical) as well as more complex association networks can be detected in both species, as discussed in more detail in a later section of this manuscript.

Despite the challenges introduced by the lack of timing information, rs-fMRI is in many ways more flexible than stimulus-related fMRI, allowing the examination of brain regions that are difficult to target with traditional sensory stimulation. A conspicuous drawback of fMRI studies in rodents has been their limitation to simple sensory studies, as cognitive experiments are typically not feasible in animals in the scanner. However, the areas of the brain involved in cognition can be examined with rs-fMRI because no response or task is required. Moreover, rs-fMRI provides greater opportunity to examine the functional organization of subcortical networks (including hippocampus, thalamus, and basal ganglia) by taking advantage of the relatively large and well-defined anatomy in rodent.

The earliest rs-fMRI studies in rodents were performed in rats (Lu et al., 2007; Pawela et al., 2008; Zhao et al., 2008; Williams et al., 2010), like most fMRI rodent studies. The larger brain of the rat as compared to the mouse makes the requirements on spatial resolution less stringent. In the past, fMRI and rs-fMRI studies were often performed under anesthetics such as α-chloralose that required intubation and careful monitoring of arterial blood gasses to maintain normal physiology. This monitoring was more demanding in mice due to the increased difficulty of arterial cannulation and their relatively small blood volumes, which limited the size and number of blood gas samples that could be obtained. Recently, however, experimenters have been moving to sedatives such as dexmedetomidine, which allow animals to breathe freely. This advance and other improvements have made it feasible to perform rs-fMRI in mouse models, paving the way for the use of transgenic models (Guilfoyle et al., 2013; Grandjean et al., 2014; Jonckers et al., 2014; Sforazzini et al., 2014; Stafford et al., 2014; Zerbi et al., 2014).

## Animal physiology and anesthesia

In comparison to human rs-fMRI, animal studies require much more attention to maintaining stable physiology in order to obtain a BOLD signal reliably linked to neuronal activity. The challenges are primarily created by the use of anesthesia, which can affect both neural activity and neurovascular coupling, which in turn impact the rs-fMRI data.

### Neurometabolic and neurovascular coupling

Functional connectivity measurements made using rs-fMRI assume that synchronized BOLD fluctuations reflect coordinated neuronal communication via neurometabolic and neurovascular coupling. Neuronal communication requires that neurons constantly manipulate the distribution of signaling molecules across their membranes, and these manipulations make up the bulk of the brain's energy requirements (Roy and Sherrington, 1890; Bélanger et al., 2011). Because the brain does not store large amounts of energy (Gatfield et al., 1966), fluctuations in brain activity are tightly coupled to energy delivery through the blood (Roy and Sherrington, 1890; Hyder et al., 2002). It is this coupling between neural activity, metabolism, and hemodynamics that forms the basis of fMRI and rs-fMRI. Specifically, the BOLD signal reflects the relative concentrations of diamagnetic oxygenated hemoglobin and its paramagnetic metabolite, deoxygenated hemoglobin. Because the BOLD signal measured by fMRI and rs-fMRI is sensitive to the local concentration of deoxyhemoglobin rather than neural activity, understanding the interaction between neural activity and the hemodynamic response is essential to interpreting the BOLD-based measures.

Stimulus-related fMRI studies have provided insight into the hemodynamic response to neural activity. After the start of the stimulation there is an anesthesia-dependent time lag before the BOLD signal increases, and the signal reaches its peak well after the stimulus has begun (Silva et al., 2000; de Zwart et al., 2005; Schroeter et al., 2014). The BOLD response to stimulation is often considered to be most closely tied to high frequency local field potentials (LFPs) but also strongly resembles multiunit activity (MUA), which has been relatively less-studied in conjunction with MRI (Spenger et al., 2000; Logothetis et al., 2001; Herman et al., 2013).

It is plausible that the same relationship holds true for the spontaneous BOLD fluctuations detected with rs-fMRI, and indeed, recent work using simultaneous rs-fMRI and electrophysiological recording to examine the neural basis of spontaneous BOLD fluctuations also found a relationship between LFPs and the BOLD signal, although it was less straightforward. High frequency LFPs, particularly in the gamma range, were tightly coupled to the BOLD signal from the same area and also to the global BOLD signal (Shmuel and Leopold, 2008; Schölvinck et al., 2010; Pan et al., 2011). On the other hand, lower frequencies like delta and theta band fluctuations seem to be more closely related to the BOLD correlation between areas (Lu et al., 2007; Pan et al., 2011). Pan et al. have shown that very slow electrical activity (below 1 Hz) also contributes to BOLD fluctuations and the correlation between areas (Pan et al., 2013). The slow oscillations can also be observed in spike trains and are closely related to changes in cerebral blood flow (Huang et al., 2014). These studies suggest that a range of processes may underlie the spontaneous BOLD fluctuations used to map connectivity. For reviews of the relationship between rs-fMRI and neural activity (see, Schölvinck et al., 2013; Keilholz, 2014).

It is also important to note that most of the simultaneous electrical recording and MRI studies performed to date examined sensory cortex. Two intriguing recently published studies show that the hemodynamic response can be quite different outside of these areas (Mishra et al., 2011; Devonshire et al., 2012), highlighting the complex nature of neurovascular coupling and the need for caution in interpreting BOLD as a surrogate for neural signaling.

### Influence of anesthesia on brain activity and the vasculature

Anesthesia is generally used in animal rs-fMRI studies to obtain stable physiological conditions. Fortunately, it has been shown in both humans and animals that the general structure of the functional networks detected with rs-fMRI transcends levels of consciousness (Vincent et al., 2007; Lu et al., 2012). Nevertheless, the alterations in neural activity and brain physiology under anesthesia should not be ignored. General anesthesia can lower brain metabolism (Gyulai, 2004) in a manner that parallels the mechanisms underlying physiologic sleep by neural suppression and network disconnection (Steriade, 1994). Anesthetic-induced unconsciousness may be a result of a thalamocortical disconnection mechanism, in which anesthetic agents compromise the natural firing patterns of thalamic network neurons and block synaptic transmission of sensory information via the thalamus (Steriade, 2001; White and Alkire, 2003). The agents used for animal studies range from mild sedatives to general anesthetics and there are substantial differences in their effects on the baseline metabolism of the brain (Maandag et al., 2007).

The BOLD signal relies on consistent neurovascular coupling to represent neural activity in a reliable manner. However, anesthesia can have side effects on animal physiology that confound the imaging results by altering vascular resistance and therefore neurovascular coupling. The processes that mediate neurovascular coupling are not completely understood, but are likely to involve moderation of adjacent arteriolar dilation and constriction by neural metabolites, such as Co_2_, K^+^, and NO. For example, arterial diameters are sensitive to the regulation of smooth muscle membrane potential through activation or inhibition of K^+^ channel activity (Nelson et al., 1990; Nelson and Quayle, 1995).

Different anesthetics can affect neurovascular coupling in different ways (Franceschini et al., 2010) and also affect functional connectivity measured with rs-fMRI (Williams et al., 2010; Pan et al., 2013; Grandjean et al., 2014). Many of the earliest fMRI and rs-fMRI studies used α-chloralose, a typical GABAergic anesthetic popular for functional imaging studies because of its relatively minor influence on neurovascular coupling and neural activity. Ueki et al. demonstrated localized metabolic activation following electrical stimulation in rats that could not be observed under halothane, a volatile anesthetic that is taken up rapidly by the brain (Butler, 1964; Ueki et al., 1992). Further work found stable and greater regional cerebral blood flow (rCBF) responses using α-chloralose than in halothane-anesthetized rats (Lindauer et al., 1993). However, the shape of the response to stimulation was similar under both anesthetic agents. This suggests that coupling between changes in neural activity and changes in blood flow can be relatively preserved despite disrupted neurometabolic coupling, and emphasizes the need for careful consideration of the effects of anesthesia on both blood flow and metabolism in order to interpret the BOLD response. For longer experiments (up to 6 h) in a study involving direct cortical stimulation, α-chloralose yielded a less stable response relative to halothane (Austin et al., 2005). Because α-chloralose depresses respiration, rats are generally artificially ventilated for the duration of the study and invasive blood gas monitoring is required, rendering the agent unsuitable for longitudinal experiments or for use in mice.

Several other GABAergic anesthetics are also used in functional imaging studies. For example, the volatile agent isoflurane is convenient, widely available and commonly used, though the issue of vasodilation and the suppression of neural activity (especially in deep anesthesia) complicate rs-fMRI studies (Fukuda et al., 2013). Volatile anesthetics tend to increase cerebral blood flow by inducing vasodilation via activation of ATP-sensitive K+ channels of arteriolar smooth muscle cell (Iida et al., 1998), though rCBF decreases can be observed in areas such as the thalamus, probably due to the predominance of neural inhibition in thalamus and thalamo-cortical pathways (Ramani and Wardhan, 2008). Rodents anesthetized with isoflurane are generally not intubated and the agent is suitable for longitudinal studies. The amount of vasodilation and neural suppression vary with the dose of isoflurane, and the extent of the functional networks detected under isoflurane exhibit a similar dose-dependence (Liu et al., 2013b). Isoflurane typically produces periodic burst-suppression rhythms across the whole brain. These highly synchronized activities obscure lower amplitude fluctuations between regions known to be functionally connected (Kalthoff et al., 2013; Magnuson et al., 2014a). This large reduction in functional connectivity is observed in humans as well under the analogous anesthetic sevoflurane (Peltier et al., 2005). In a dose dependent manner, between 78% (light anesthesia) and 98% (heavy anesthesia) of the motor cortex voxels that were significantly correlated under the awake state become disconnected.

Rather than general anesthesia, sedation is often preferable for functional neuroimaging studies. Medetomidine and its active enantiomer, dexmedetomidine, are α2-adrenergic agonists that induce sedation (Weber et al., 2006; Kalthoff et al., 2013). Medetomidine has been used in the clinic with many benefits including anxiolysis, blood pressure stabilization, analgesia, anesthetic sparing effects and sedation without respiratory depression or significant cognitive impairment (Cormack et al., 2005; Rozet, 2008). In recent years, dexmedetomidine (or medetomidine) was introduced to rodent fMRI studies. The α2-adrenergic agonist induces sedation through endogenous sleep pathways by its action on the locus ceruleus (which has the highest presynaptic α2-adrenergic receptor concentration) that in turn decrease the afferent input to the thalamus and thus decrease thalamic activity (Correa-Sales et al., 1992; Nelson et al., 2003; Bonhomme et al., 2008). In both human and animal studies, dexmedetomidine administration causes a clear decrease in global CBF (Zornow et al., 1990; Prielipp et al., 2002; Drummond et al., 2008; Fukuda et al., 2013), which some suggest arises from cerebral vasoconstriction (Zornow et al., 1990; Prielipp et al., 2002). The cerebrovascular effects of dexmedetomidine are primarily mediated via activation of α2-adrenergic receptors and result in increased vascular resistance via vasoconstriction. Dexmedetomidine sedation does not require intubation and can be reversed by an injection of atipamezole, making it suitable for longitudinal studies. It has become popular for rs-fMRI studies as it lacks the dose-dependent vasodilation and neural suppression that occur under isoflurane. A potential problem is the stability of the preparation for long studies. Pawela et al. suggested that the initial dose of medetomidine be tripled after the first couple of hours to maintain sedation (Pawela et al., 2009). Magnuson et al. also found that the characteristics of the spontaneous BOLD fluctuations and functional connectivity changed over time while rats were sedated with medetomidine (Magnuson et al., 2014b). Clearly there is a need for further studies on the exact effects of prolonged administration of (dex-) medetomine in functional imaging studies.

While the variability of neurovascular coupling and metabolic state under different anesthetics is a potential confound for rs-fMRI studies, fortunately the influence of anesthesia appears to modulate neurovascular coupling instead of completely abolishing neurovascular coupling (Pan et al., 2013). Nonetheless, caution should apply particularly during transitions between drugs or dose levels because the effects of the agent on the vasculature have been known to cause transient instabilities in hemodynamic parameters. Therefore, it may be necessary to establish a waiting period near dose or drug transitions to ensure that a stable state is reached.

Although anesthesia is preferred in most imaging experiments to ensure that animals remain motionless, fMRI in awake rodents has been developed and reported by a few groups (King et al., 2005; Zhang et al., 2010; Becerra et al., 2011; Febo, 2011). These studies facilitate an examination of the alterations in neurovascular coupling induced by anesthesia. For example, hemodynamic responses were longer in latency, from 2 s in awake to 4 s in anesthetized rats, and exhibited wider response functions, from 0.6 s in awake to 2.5 s in anesthesia (Martin et al., 2006). Although awake animal models are closer to human studies performed in conscious state, major challenges remain. Most studies using awake rodents acclimate the animals to the stress of restraint over several days in an effort to minimize the acute stress induced by the scanning session (Febo, 2011). However, the acclimation process itself may have an effect as it could be considered a chronically stressful situation; indeed, certain groups have used restraint as an effective approach for developing a depression model in rodents (Willner et al., 1995; Stepanichev et al., 2014).

### Maintaining stable physiology during rs-fMRI

Resting state studies assume a stable physiological condition for all subjects (whether human or animal), since changes related to physiological variations such as temperature are not generally of interest. Relative to stimulation-related or evoked activity studies, rs-fMRI analysis examines a relatively longer time period, i.e., several minutes in a resting study vs. seconds or sub-second periods in an evoked event study. During the longer period of data acquisition, the risk of variation in animal's physiological state generally increases. Since, the spontaneous neural events that occur during rs-fMRI are not predictable in a time-locked fashion, the method of noise removal by averaging used in event-related designs is not possible and physiological fluctuations that provide unwanted contributions to the rs-fMRI signal can alter correlation between areas.

**Figure 2:**
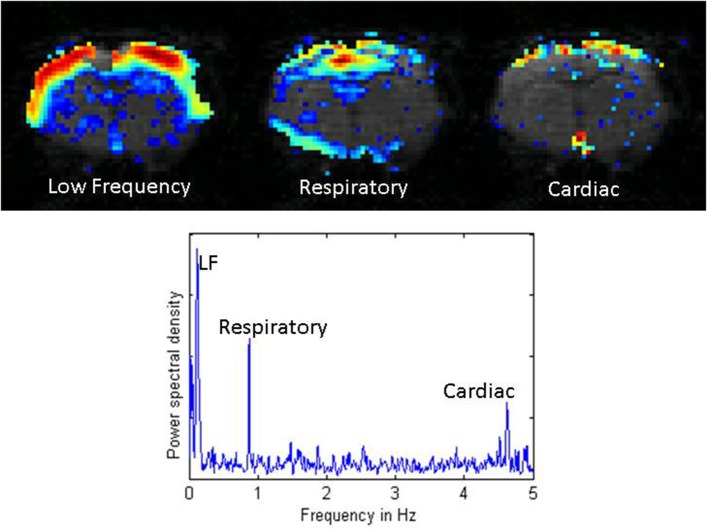
**Distribution of power for low frequency fluctuations, respiratory noise, and cardiac noise from a single coronal slice in a rat imaged with a TR of 100 ms**. The low frequency fluctuations exhibit high power across the entire cortex. Respiratory effects localize near the midline, the ventricles, and the surface of the brain. Cardiac effects are primarily seen along the surface of the brain and at the base of the brain. The power spectral density plot below shows the low frequency range (< 0.2 Hz), the respiratory peak at ~1 Hz, and the cardiac peak at ~4.7 Hz. Adapted from Williams et al. MRI (2010).

As a first step toward obtaining stable physiological conditions and ensuring reproducible experimental conditions, experimental groups of animals should be consistent in strain, gender, age, weight, housing condition, food and lighting cycle, and so on (Hildebrandt et al., 2008). Furthermore, physiological parameters should be monitored and controlled (to the extent possible) during an experiment. These physiological parameters include anesthetic dose, body temperature, blood oxygenation level, and respiratory and cardiac rates. Fluctuations in body temperature can contribute to drifts in the BOLD signal baseline, even when the temperature changes are within physiological ranges (Vanhoutte et al., 2006). Respiratory and cardiac cycles are known to contribute to the rs-fMRI signal and can introduce unwanted correlations (Wise et al., 2004; Birn et al., 2006; Shmueli et al., 2007) (Figure 2). Ideally, these contributions can be removed in post-processing if the cardiac and respiratory cycles are recorded and time-locked to rs-fMRI acquisition (Hu et al., 1995; Glover et al., 2000). For the respiratory cycle, the most common recording device is a pressure-sensitive balloon placed under the animal's chest and abdomen. For the cardiac cycle, electrocardiography is a logical choice but often proves unreliable in practice due to interference from the gradients during image acquisition. An alternative is a pulse oximeter, usually applied to one of the animal's hind limbs. This device does not record the entire cardiac cycle, but does give a time course of the heart rate and blood oxygenation level. For studies that involve comparing different groups of animals, showing that the heart rate and blood oxygenation levels are comparable across groups and stable over the course of the experiment greatly increases confidence that any effects on functional connectivity are not due to these basic physiological processes.

## Data acquisition

### Hardware

Most rodent fMRI experiments are performed on specialized high field animal scanners. While a typical field strength for a rs-fMRI exam in humans is 3 T, in rodents the field strength is commonly 7, 9.4 or 11.7 T, but the magnet bores are smaller (20–30 cm) (Lu et al., 2007; Pawela et al., 2008; Zhao et al., 2008; Williams et al., 2010; Kalthoff et al., 2013; Hyde and Li, 2014). The increased field strength improves the signal to noise ratio, an important step toward obtaining high quality data from the small rodent brain. Spatial resolution must be much higher than in human subjects to obtain comparable coverage of the brain's structures. Strong gradients help to achieve the necessary resolution. fMRI studies in rodents also benefit from stronger, higher order shims to minimize distortion, since echo-planar imaging (EPI) is commonly used for data acquisition. Whole brain studies are often performed with a transmit/receive head coil similar to those used in humans; more localized studies of selected brain areas often combine the head coil for transmission with a small surface coil to receive the signal from the areas of interest (Lu et al., 2007; Pawela et al., 2008; Zhao et al., 2008; Williams et al., 2010).

### Acquisition parameters and contrast

As in human rs-fMRI studies, most rodent studies employ fast imaging sequences to image the brain on the order of once per second (Lu et al., 2007; Pawela et al., 2008; Zhao et al., 2008; Williams et al., 2010). EPI is the most common sequence, generally employed in the gradient-echo based form. A spin-echo based EPI approach is sometimes useful to minimize susceptibility artifacts but also reduces sensitivity to the BOLD signal (Majeed et al., 2009b). While human studies are moving to multiband sequences that allow whole brain imaging with subsecond TRs (Feinberg et al., 2010; Moeller et al., 2010), these sequences have yet to penetrate to the animal imaging community, partly because multi-channel systems are less widely available. A typical rs-fMRI study in rats has an in-plane spatial resolution of 200–400 microns, a slice thickness of 1–2 mm, a repetition time (TR) of 0.5–2 s, and a matrix size of 64 × 64 to 128 × 128. Scan lengths range from approximately 200–1000 images (Zhao et al., 2008; Hutchison et al., 2010; Williams et al., 2010; Kalthoff et al., 2013; Hyde and Li, 2014). Echo times (TEs) are shorter than for comparable human experiments to maximize BOLD contrast at high fields while minimizing signal loss. Most images are acquired in the coronal plane, possibly due to the predominance of this orientation in atlases and histological studies, but some groups have used other orientations to better characterize particular networks of interest (Hudetz et al., 2015).

In addition to BOLD contrast, rs-fMRI studies can also be performed with contrast based on cerebral blood volume (CBV) or CBF. CBV contrast is commonly achieved by the injection of ultrasmall iron oxide particles and can provide greater sensitivity to stimulus-related changes than BOLD (Magnuson et al., 2010; Schölvinck et al., 2010; Sforazzini et al., 2014). CBF contrast is often obtained using a version of arterial spin labeling in humans (Chuang et al., 2008; Mayhew et al., 2014) but in rodents, optical methods have typically been preferred (Bruyns-Haylett et al., 2013; Bergonzi et al., 2015).

### Assessment of data quality

A number of factors can influence data quality in practice, which come from either animal physiology or technical aspects of the scan. Data quality assessment is therefore a necessary step prior to further data analysis. For the animal physiological aspects, reviewing animal physiological recordings over the period of rs-fMRI scans and ensuring that the animal is in a stable and normal range in basic physiology is extremely important to ensure reproducible results. The quality of the imaging data should also be assessed. If any “spikes” or short-lived high amplitude changes are detected in a resting time course, they may be indicate noise due to motion or other uncontrolled technical issues during the scan. Thus, it is recommended to begin with reliable data that has passed quality assessment before pursuing further analysis.

## Preprocessing

**Figure 3:**
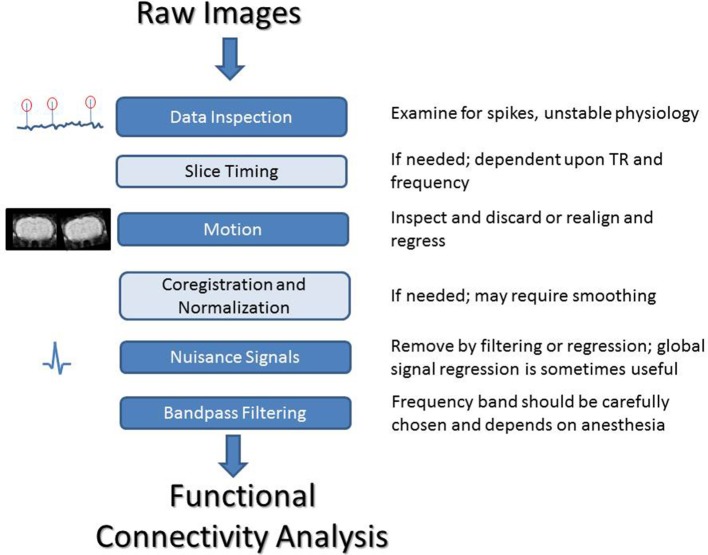
**A summary of processing steps for rodent rs-fMRI**. After acquisition, it is critical that both the image time course and the recordings of physiological parameters are closely examined. Slice timing correction can be performed if needed, depending on the frequency range of interest and the TR of the scan. All data should be inspected for motion and either corrected or discarded. For group studies, co-registration and normalization can be used to align images to a common space. Nuisance signals should be handled carefully. The frequency range for filtering can be chosen based on previous work showing coherence between BOLD and LFP for different anesthetics or empirically based on an examination of the power spectrum of the BOLD fluctuations. Standard frequency ranges from human studies are not necessarily applicable to animal work and may result in the loss of a large amount of information.

Human rs-fMRI data typically undergoes extensive preprocessing prior to further analysis. Motion correction, slice timing, corrections for physiological noise, spatial and temporal filtering, and some type of normalization are commonly applied (Murphy et al., 2013). While many of these steps are also appropriate for rodent data, some changes are necessary to obtain optimal results. Many software packages used for fMRI and rs-fMRI preprocessing in human studies, such as Statistical Parametric Mapping (SPM) and Analysis of Functional NeuroImages (AFNI) (Cox, 1996), can also be used for rodent studies, although modification to the data format and parameters such as field of view may be necessary, and many labs use their own code instead. Here we summarize common preprocessing considerations for rodent rs-fMRI studies (Figure 3).

### Motion correction

Slight motions of the head are common in human rs-fMRI studies and can lead to large-scale correlation. Even miniscule shifts in position can result in a change in tissue type within voxels and changes in slice positions. In addition to the direct effects of motion, spin-history effects can also arise, though corrections for this do exist (separate from realignment) (Friston et al., 1996; Muresan et al., 2005). Motion correction by rigid-body realignment is typically applied to human rs-fMRI data before further analysis, often combined with a “scrubbing” procedure to remove instances of high motion (Power et al., 2012). In rodent studies, however, animals are typically restrained in a stereotaxic device with ear bars and a tooth bar. The use of anesthesia further minimizes movement. For these reasons, the amount of motion in a rodent rs-fMRI study is typically much less than for a human rs-fMRI study. It is known that motion correction can actually cause artificial correlation if improperly applied (Grootoonk et al., 2000), so many groups that work with animals choose to inspect the motion time courses for each scan and discard any that exhibit significant motion. Realignment is also not well-suited for data that covers only a portion of the brain.

### Slice timing correction

The acquisition of brain slices is typically interleaved during the TR to minimize interference, which means that adjacent brain areas may be imaged at different times. Slice timing correction addresses this issue and can be accomplished using temporal sinc interpolation. For a human study with a 2 s TR that is low-pass filtered with a cutoff at 0.1 Hz, the maximum phase difference between slices would be approximately one-fifth of a cycle. Whole-brain studies in rodents often are performed with a similar TR and a cutoff of 0.1 Hz or even higher, and slice timing correction may prove beneficial for these studies. For studies with a limited number of slices and a short TR, however, the minor phase differences may not be of concern, especially as the use of slice timing correction can propagate any artifacts in one image throughout the time series because of the sinc interpolation.

### Segmentation, coregistration, and normalization

As in human studies, high resolution anatomical images are commonly acquired along with rs-fMRI data. The EPI images used for rs-fMRI typically have poor spatial resolution, low anatomical contrast, and suffer from distortion, so it can be advantageous to acquire an anatomical scan to better delineate structures within the brain. The anatomical and EPI images are nominally registered due to common positioning within the scanner, but in practice more sophisticated registration methods can be required, due to blurring and warping in the EPI images. Segmentation based on anatomical images is widely used in human studies to separate the brain into gray matter, white matter, and cerebrospinal fluid (CSF). In rodents, however, the use of segmentation is not common, partly due to the much lower volume of white matter present.

To compare across subjects or groups, it is necessary to map functional networks to a common space. In humans, this is generally accomplished through the use of atlases (MNI, Tailarach). Templates for aligning images to atlases are also available for rodents. Examples include the Paxinos atlas (Schweinhardt et al., 2003), the Waxholm atlas (Papp et al., 2014) and others (Schwarz et al., 2006; Lu et al., 2010; Valdés-Hernández et al., 2011). Fitting an individual image to an atlas is usually accomplished using non-linear transformations that fit functional images to a template, whether by matching anatomical landmarks to the template atlas or using volume- and surface-based methods. Group analysis can then be performed (Kalthoff et al., 2013; Liang et al., 2013).

In general, anatomical variability is much lower in the rodent strains used in laboratory work than it is in humans. Instead of atlases, many groups create an average or typical image for their study and register the remaining data to it (Hudetz et al., 2015; Jonckers et al., 2014). Alternatively, data may be analyzed on an individual level then pooled to identify group level characteristics (Zhao et al., 2008; Hutchison et al., 2010; Williams et al., 2010).

### Controlling for nuisance signals

Nuisance signals are artifactual signals unrelated to neural activity that can influence many or all voxel time series. They can manifest as structured noise that introduces spurious correlations in the data and can influence results. These signals can arise from respiration and the cardiac cycle, among other things (Murphy et al., 2013). Ideally, if these cycles were recorded during the scan, their contributions can be removed on a voxel-by-voxel basis using regression methods (Glover et al., 2000). It is also sometimes possible to remove the primary contribution of respiration and cardiac pulsation directly based upon frequency. Typical heart rates for rats depend on the age and the anesthetic used, but are around 4–5 Hz. Respiratory rates are closer to 1 Hz. With most animal scanners it is possible to sample a single slice rapidly enough to resolve the primary spectral peaks from these processes. Using a 100 ms TR, Williams et al. found that in general, respiratory contributions peak near the midline and brain surface, while cardiac contributions are greatest near the base of the brain (Williams et al., 2010). However, the sampling rates required to resolve the physiological cycles typically preclude the whole brain coverage needed for many studies.

In human studies where physiological cycles are not recorded, proxies must be used. Global signal regression is sometimes employed for this purpose, although its use is controversial. It promotes more focal correlation patterns (Fox et al., 2009) but can also introduce artifactual anti-correlations (Murphy et al., 2009) or reduce sensitivity to true neural signals (Schölvinck et al., 2010). In rodents, global signal regression has sometimes been used to minimize the effects of varying doses of isoflurane (Liu et al., 2013b). As an alternative to global signal regression, human studies sometimes use the regression of the white matter and CSF signals. However, these areas are small in volume in the rat and it can be difficult to obtain a clean signal.

Because of the difficulty in accurately correcting for physiological noise based on image data alone, physiological cycles should be recorded in rodent rs-fMRI studies if at all possible. This also allows the experimenter to ensure the stability of the animal throughout the experiment. In the rat, motion can be linked to the respiratory cycle, a finding attributed to the motion of the thorax within the magnetic field resulting in frequency offsets and apparent shifting of the brain in the phase encode direction (Kalthoff et al., 2011). Careful application of motion correction and regression of motion and respiratory parameters can minimize the effect on further analysis.

### Filtering

In humans, BOLD fluctuations are typically filtered with a high pass cutoff at 0.08–0.1 Hz. However, recent advances in fast imaging have shown that much higher frequencies can contribute to functional connectivity (Lee et al., 2013). This is in line with studies in rodents beginning with some of the earliest rodent work that have shown that the BOLD fluctuations have substantial power in frequencies above 0.1 Hz and that these frequencies contribute to functional connectivity (Majeed et al., 2009a; Magnuson et al., 2010; Williams et al., 2010). The precise range of frequencies that are coherent with neural activity varies for different anesthetic agents (Pan et al., 2013) and so it is important to examine the power spectrum for each study individually, to determine that appropriate filters are used.

## Data analysis and presentation

Seed-based correlation is the approach used in many of the earliest functional connectivity studies (Biswal et al., 1995) and is still popular today, particularly for hypothesis-driven studies. A typical implementation involves selecting a seed that may cover a few voxels or an entire functional area of the brain. The correlation coefficients between its average time series and the time series from every other voxel in the brain are calculated. Significant correlation can be identified and mapped spatially, with maps combined for group analysis using registration to an atlas or average image, or the average correlation in a second target region of interest can be measured. Seed-based correlation is relatively easy to interpret but is not well suited to exploratory analysis. It remains very popular for rs-fMRI studies in rodents, particularly those driven by area-based hypotheses (Lu et al., 2007; Pawela et al., 2008; Zhao et al., 2008; Williams et al., 2010; Keilholz et al., 2013; Hyde and Li, 2014).

Independent component analysis (ICA) is a form of blind source separation that is growing more and more popular for human and rodent rs-fMRI studies (Hutchison et al., 2010; Lu et al., 2012; Jonckers et al., 2014). ICA searches for a set of minima in the cost function describing signal independence to provide a data-driven technique for identifying networks of functional connectivity. Despite its growing use in functional neuroimaging, challenges remain in the use of ICA for rs-fMRI analysis. Depending on the number of components used, the results can give localized nodes in single areas or large scale networks distributed across the brain. The resulting components are unranked, making identification of networks somewhat ambiguous. Nevertheless, ICA has proven useful in a wide range of rs-fMRI studies.

Extensions to the basic functional connectivity analysis have been developed in human research and are now being translated to the rodent. In graph theory, for example, networks are represented as a graph with the nodes comprised of brain regions or image voxels and their interconnections are determined by the correlation strength between each pair of regions (Schwarz et al., 2012). The technique is growing in popularity in human rs-fMRI as a method for summarizing the immense quantity of data obtained in each scan, and holds equal promise for rodent rs-fMRI (Keilholz et al., 2010; Stafford et al., 2014).

Dynamic analysis is another recent development in the field of rs-fMRI. Until recently, most rs-fMRI studies in both humans and animals assumed that the relationships between brain areas were maintained for the entire length of the scan (5–10 min). Recent work in multiple species has shown that fluctuations in correlation between areas occur over much shorter timescales (Chang and Glover, 2010; Hutchison et al., 2013a; Keilholz et al., 2013). It is hypothesized that some portion of these fluctuations are related to changes in the coordination of neural activity, though research has shown that some variability can also arise from inherent signal properties (Handwerker et al., 2012; Keilholz et al., 2013). The dynamic approach offers the potential to examine “brain states” linked to cognition or altered by dysfunction.

Data presentation is relatively straightforward in the human, where most analysis is performed on group maps in a common space. When rodent data is registered to an atlas or standard image, the same methods can be used. For data with limited brain coverage, however, this is often not feasible. In this case, rather than show a single representative rat, it is recommended that a sample or (if possible) the entire set of data be shown so that readers can assess the variability across scans and/or subjects. We also urge authors to consider showing the raw EPI data. Especially for rodent studies, regions of interest are often small and near areas of susceptibility artifact. Showing the EPI images (rather than anatomical scans) beneath functional connectivity maps allows the reader to evaluate potential effects of image artifacts and blurring.

## Functional networks

A rough estimate of the spatial extent of functional brain regions (for example, those involved in processing a specific stimulus) has been obtained with fMRI studies. Two-dimensional surface microscopy of the blood flow response to activation of a single whisker barrel cortex demonstrates that when a single cortical column is activated, blood flow increases within the associated venous unit and its upstream artery (Berwick et al., 2008). In high-resolution fMRI studies, activation can be observed at the level of a single column or as a function of lamina (Yang et al., 1996, 1998; Silva and Koretsky, 2002). While the ultimate resolution of rs-fMRI is unknown, there is evidence that connectivity differences at the level of columns may be discerned. Using a suite of customized hardware and software, Hyde and Li acquired BOLD venography and echo-planar images (EPI) over the rodent cortex with 200 μm^3^ voxels (Hyde and Li, 2014). At this scale, layers V and VI are shown to produce the most extensive FC networks. Bilateral connections were observed between seed regions in these layers and a sparsely distributed collection of voxels in the primary and secondary somatic and motor cortices (S1, M1, S2, and M2, respectively). Layer II-III shows consistent local connectivity with a tight group of voxels in layers V and VI of the same cortical column, but little inter-regional connectivity.

While specificity can be observed at the laminar level, connectivity is often examined on a larger scale. Most rs-fMRI studies effectively average the hemodynamic response within multiple cortical layers and/or columns to produce FC maps that are likewise more spatially extended (Kriegeskorte et al., 2010). For example, when a seed is chosen in unilateral SI, the resulting correlation extends beyond the seed region on the ipsilateral side and is present in a roughly equal area on the contralateral side (Pawela et al., 2008; Zhao et al., 2008; Williams et al., 2010).

Much is known about the anatomical connectivity of rodent brains due to the volume of tracer studies performed in these species. Comparison of these known patterns of structural connectivity to functional connectivity make it clear that functional networks require intermediary connections but do not utilize all tracts to the same degree. For example, left and right primary somatosensory cortex are directly connected via the corpus callosum and as expected exhibit strong functional connectivity. Left and right caudate putamen, however, are not directly connected but still exhibit strong functional connectivity (Williams et al., 2010; Nasrallah et al., 2014b). Similarly, transection of the corpus callosum reduced functional connectivity between left and right SI but did not eliminate it (Magnuson et al., 2014a).

As in human studies, a number of functional networks are consistently observed across subjects in rodents. These networks typically match alternatively defined (histology, lesion studies, electrophysiological) physiological and anatomical parcellations. Bilateral networks, such as those covering somatomotor, visual, hippocampal and other areas are consistently isolated. Spatially distributed networks corresponding to interoceptive and exteroceptive networks have also been observed. In the following sections, we will discuss the two of these FC networks.

### The somatomotor network

The somatosensory network has been the primary target for studies that attempt to elucidate the neural and hemodynamic underpinnings of the rs-fMRI signal (Lu et al., 2007; Pan et al., 2011, 2013). The first evidence demonstrating rs-fMRI-based FC showed that the areas activated by a bilateral finger tapping task are functionally connected to one another during rest (Biswal et al., 1995). Analogous experiments have been performed in the rat. In this case, electrical stimulation of the radial nerve acted as the “task” to which functional connectivity patterns were contrasted (Pawela et al., 2008). Stimulation of the combined afferent/efferent fiber bundle produced activation patterns that included regions in the primary and secondary motor cortex(M1, M2), the primary and secondary somatosensory cortex (S1, S2), and the somatomotor thalamus. Seed-based connectivity analysis conducted from each of these seed regions produced FC maps closely overlapping the activated regions. Similar patterns of functional connectivity created using seed-based techniques were observed by other groups as well (Lu et al., 2007; Zhao et al., 2008; Williams et al., 2010).

ICA FC maps of rodent resting state fMRI data demonstrate that M1 and S1 are carved out into a single network out of the remainder of the brain when the brain is segmented into between 10 and 20 independent networks (Becerra et al., 2011; Kalthoff et al., 2013). If, however, the brain is segmented into more components, 100 or so, FC networks are carved into smaller units whose composition differs between species. In humans, these smaller networks often continue to straddle the central sulcus, maintaining their bilateral associations while becoming reduced in their dorsal to lateral expanse (Kiviniemi et al., 2009; Abou Elseoud et al., 2011). In the case of the rodent, these more local networks form lateralized domains along the cortical ribbon (Jonckers et al., 2011). These smaller rodent networks isolate each region, M1, M2, S1, and S2, into independent regions, albeit with some overlap.

### Default mode network (DMN)

The DMN is a collection of distributed brain regions which includes the midline frontal cortices, integrative midline cortices along the cingulate, visual association cortices in the parietal lobe, auditory association cortices in the temporal lobe, and the hippocampus. It was first observed in positron emission tomography and fMRI studies in humans as the set of regions whose activity is reduced upon the onset behavior directed toward external goals (Raichle et al., 2001). Subsequent studies in non-human primates and rodents have confirmed its consistent appearance in mammalian FC networks (Fox et al., 2005; Buckner et al., 2008; Mantini et al., 2011; Upadhyay et al., 2011; Lu et al., 2012). Despite the association of the DMN with internally-oriented mental activity, it is present during sleep, under anesthesia, and in vegetative cases, demonstrating that it is not an indication of conscious activity. The DMN has been implicated in cognitive performance and is altered in a wide range of psychiatric and neurological disorders in humans. Its presence in the rodent will allow multimodal studies that provide new insight into the function and dysfunction that underlie the alterations observed in humans.

## Applications

### Neurophysiology of functional connectivity and neurovascular coupling

One of the primary contributions of animal rs-fMRI studies to the field so far has been to provide insight into the neural mechanisms underlying the BOLD signal correlations (Lu et al., 2007; Shmuel and Leopold, 2008; Schölvinck et al., 2010; Pan et al., 2011, 2013; Thompson et al., 2013b,c, 2014). This has been facilitated by the ability to combine standard MRI techniques with more invasive measures that are sensitive to neural activity. Using epidural electrodes and rs-fMRI in separate groups of rats, a pioneering study by Lu et al. showed that correlations in band-limited power (BLP) in the delta frequency band changed in the same way as BOLD correlations with increasing depth of α-chloralose anesthesia (Lu et al., 2007). A subsequent study that acquired simultaneous rs-fMRI and microelectrode recording in non-human primates found that gamma band power was most strongly linked to the spontaneous BOLD fluctuations from a single site (Shmuel and Leopold, 2008). These somewhat contradictory results motivated another simultaneous rs-fMRI and microelectrode recording experiment, this one involving bilateral sites in the rodent. Pan et al. found that while gamma BLPs were most tightly correlated to the local BOLD signal, low frequency (delta and theta) BLP correlation was most predictive of changes in BOLD correlation as a function of anesthesia depth (Pan et al., 2011), confirming both previous studies and suggesting that anesthesia may affect the frequency of neural activity that contributes most strongly to functional connectivity. A later experiment from the same group showed that electrical activity in the same low frequency range as the BOLD fluctuations themselves (< 1 Hz) is directly linked to the rs-fMRI signal and contributes to interhemispheric BOLD correlation (Pan et al., 2013).

Animal models have also proved important in understanding the effect of the baseline brain state upon the BOLD signal correlations. Anesthetics affect neural activity, metabolism, and vascular tone, resulting in differences in the BOLD correlations observed (Williams et al., 2010; Kalthoff et al., 2013; Liu et al., 2013a; Grandjean et al., 2014; Jonckers et al., 2014; Nasrallah et al., 2014a). These changes in baseline also affect the response to stimulation (Maandag et al., 2007).

Most of the multimodal studies in animal models described above were performed in sensory cortex and confirmed a neural origin for the BOLD fluctuations. This finding provides important support for the use of rs-fMRI to study the brain in humans and aids in interpreting the results. However, recent work involving multimodal measurements from other parts of the brain indicates that neurovascular coupling depends strongly on brain region. Mishra et al. found that hemodynamic signals from the caudate putamen decreased despite an increase in LFP and spiking activity in a rat model of epilepsy (Mishra et al., 2011). Another study by Devonshire et al. demonstrated non-linear neurovascular coupling along the whisker-to-barrel cortex pathway, showing that even areas involved in the same sensory system do not necessarily exhibit the same neurovascular coupling (Devonshire et al., 2012). These studies make clear the need for further multimodal studies in animals to better characterize the neurovascular coupling across the brain to facilitate interpretation of the data acquired in humans.

### Validation of network dynamics

The recent move toward dynamic analysis of rs-fMRI data has proved both valuable and challenging (Hutchison et al., 2013a). While network dynamics have shown sensitivity to alterations caused by disease (Li et al., 2014; Miller et al., 2014; Rashid et al., 2014), the changes in correlation over time are also related in part to inherent properties of the signal (Handwerker et al., 2012; Keilholz et al., 2013). Multimodal studies in animals have provided key information about the neural basis of these network dynamics (Keilholz, 2014).

The first observation of non-stationarity in rodents was reported by Majeed et al., who found reproducible, quasiperiodic spatiotemporal patterns of BOLD fluctuations in the anesthetized rat (Majeed et al., 2009b). The subsequent development of an autoregressive pattern finding algorithm demonstrated that these patterns are present in humans as well (Majeed et al., 2011). Further work with simultaneous imaging and electrophysiological recording in the rat suggests that these patterns are linked to infraslow electrical activity throughout the brain (Pan et al., 2013; Thompson et al., 2013c, 2014).

Most studies in humans and animals have examined network dynamics using a sliding window approach (Chang and Glover, 2010; Hutchison et al., 2013b; Keilholz et al., 2013; Kiviniemi et al., 2011). Using simultaneous rs-fMRI and microelectrode recording in rats, Thompson et al. showed that BOLD sliding window connectivity at least partially reflects underlying LFP coordination, particularly in the theta, beta and gamma frequency bands (Thompson et al., 2013b). Because the sliding window correlation appears to have a different neural basis than the quasiperiodic patterns, these studies raise the possibility that the rs-fMRI signal contains potentially separable information about distinct types of brain activity.

### Stroke, ischemia, and plasticity

Resting state MRI studies in humans have shown that brain pathologies produce, and may be characterized, by altered resting-state network behavior (Pratt et al., 2012; Rubinov and Bullmore, 2013). As the use of rs-fMRI as a tool for the evaluation and diagnosis of pathological connectivity grows, researchers are increasingly turning to rodent models to understand the basis of the alterations in connectivity that are observed. One of the most valuable aspects of rs-fMRI is that it can be applied equally to rodents and humans, allowing direct cross-species comparisons and facilitating interpretation. This is particular valuable in the context of the genetic manipulations and controlled perturbations that are possible in small animals.

Rodent models of stroke are well-developed and have been characterized extensively using multiple analytical techniques. The non-invasive nature of rs-fMRI makes it an attractive tool for longitudinal studies that address questions involving recovery of function. Resting state MRI has been successfully applied in rats toward understanding how the brain reorganizes after a stroke to recuperate function (van Meer et al., 2010; van Meer, 2011). The authors found that complete recovery of function is only associated with functional reorganization of the areas surrounding the ischemic lesion. This is as opposed to an alternate strategy in which the brain attempts to reroute lost functions by reorganizing the contralateral site. Recovery outcomes across the board were enhanced when inter-hemispheric functional connectivity remained strong. These findings corroborate with longitudinal studies among human stroke patients (Xu et al., 2014) to establish prognostic heuristics and point to possible therapeutic intervention, such as transcranial magnetic stimulation, for the enhancement of post-stroke recovery (Hoyer and Celnik, 2011; Pinter and Brainin, 2013).

Resting state MRI has also been used to characterize plasticity in response to a peripheral rather than cerebral injury (Pawela et al., 2010). Two weeks after transection of the four nerves of the brachial plexus, intrahemispheric somatomotor connectivity was mostly preserved, while interhemispheric connectivity was reduced. Changes were also seen in the connectivity of thalamic nuclei, while an unaffected system (visual) remained unchanged. Resting state MRI in rodent models thus offers a method to identify functional changes associated with acute and chronic alterations of regional innervation and plasticity. rs-fMRI is well suited to these types of studies, as it allows whole brain information to be acquired non-invasively for longitudinal experiments.

### Neurodegenerative disorders

One of the strengths of rs-fMRI in rodent models is the ability to combine the non-invasive measurements used in humans with genetic manipulations to provide insight into alterations that occur in neurodegenerative orders. A recent study by Zerbi et al. used rs-fMRI to examine apoE4 and apoE-knock out (KO) mice in conjunction with immunohistochemistry, perfusion measurements, and diffusion weighted MRI (Zerbi et al., 2014). Compared to wild-type, both strains of mice showed deficits in functional connectivity. The results suggested that the changes in functional connectivity, which are also observed in human apoE-ε4 carriers, may be related to vascular risk and the efficacy of apoE at modulating synaptic density. This study is an excellent example of how rs-fMRI can be used across species, and especially in conjunction with transgenic models, to provide insight into the neurophysiological basis of brain disorders.

### Epilepsy

Epilepsy is one of the rare cases in which invasive recordings can be obtained in human subjects, as electrode implantation and recording is commonly performed prior to surgery to localize the epileptic focus. This offers the exceptional possibility of directly comparing measurements of localized neural activity as well as rs-fMRI across species. To this end, Nir et al. examined electrical activity in presurgical patients and identified slow (< 0.1 Hz) oscillations in gamma LFP power and neuronal firing rates that were correlated across hemispheres (Nir et al., 2008), in general accordance with findings linking BOLD fluctuations and gamma power in non-human primates (Shmuel and Leopold, 2008). A complementary study by He et al. that examined different arousal levels found consistent correlation between slow cortical potentials across wakefulness, slow wave sleep, and rapid eye movement sleep (He et al., 2008). Gamma power exhibited a similar correlation structure except during slow wave sleep. These types of invasive studies are adding new pieces to the puzzle of the complex relationship between neural activity and BOLD signal correlations.

Resting state fMRI and electrical recording are also utilized in translational epilepsy research. One very nice example is described by Mishra et al. (2013), who examined a rat model of absence seizures with accompanying spike wave discharges and found increased interhemispheric connectivity, particularly in areas involved in the seizures. These findings recapitulate the increased interhemispheric connectivity found in children with absence epilepsy even in times between seizures (Bai et al., 2011).

### Stress and depression

While it is understandably difficult to reliably model human conditions such as stress, depression or anxiety in animal models, there are well-established procedures for reproducing at least some of the outward effects observed in humans. Using a chronic immobilization model to induce stress, Henckens et al. found that the rats developed increased functional connectivity within sensory and default mode networks (Henckens et al., 2015). Importantly, the changes in functional connectivity were much greater than any anatomical changes, emphasizing the need for this type of functional study. In a different approach, Liang et al. used a single exposure to predator odor to mimic the effect of a traumatic experience in rats (Liang et al., 2014). Increased anxiety and a decrease in amygdala-medial prefrontal cortex connectivity persisted for at least 7 days after the stressor.

The effects of stress during the prenatal period have also been examined in rats (Goelman et al., 2014). Rat pups from stressed mothers exhibited depressive-like behavior and altered functional connectivity in the dopaminergic system. When the mothers were given a monoamine oxidase inhibitor, the pups' behavior was restored to that of control animals and functional connectivity normalized.

Researchers have also begun to use rodent FC models to address psychological disorders such as depression. Recent findings in a genetic rat model of major depression closely parallel findings in similar humans studies (Williams et al., 2014), suggesting that further work in these models may help to elucidate mechanisms underlying the disorder and possible therapeutic interventions.

### Drug studies

In animal models, rs-fMRI is playing an important role in assessing the effects of drugs used to treat different disorders. These studies can be difficult due to the interacting effects of anesthesia and drug, but careful experimental design can allow some insights to be gained. For example, Gass et al. showed that haloperidol (an antipsychotic and dopamine D2 antagonist) reduced connectivity in areas that were strongly associated with ascending dopaminergic projections (Gass et al., 2013). These types of studies can aid in understanding the therapeutic mechanisms employed by drugs and may lead to more specific treatments.

Resting state MRI has also been used to understand the causes and consequences of drug abuse. One study in rats that were trained to self-administer cocaine and then underwent enforced abstinence showed that functional connectivity between the nucleus accumbens and dorsomedial prefrontal cortex was decreased (Lu et al., 2014). Individual differences in this connectivity were linked to the level of escalation in cocaine administration. These findings match well with similar deficits observed in abstinent human drug users. These examples serve to illustrate the flexibility of rodent fcMRI models toward the study of a multitude of human health-related conditions.

## Conclusion and future directions

Resting state functional connectivity offers a new paradigm via which to understand how the brain utilizes systemic communication to produce its many states. Rodent models have played an important role in understanding the neural basis of BOLD correlations and are likely to continue to do so. In addition, rodent rs-fMRI studies are rapidly expanding into the wide realm of animal models of brain disorders, a welcome development that will facilitate the transfer of knowledge between rodent and human research. The non-invasive nature of rs-fMRI allows it to be applied across species, so that biomarkers developed from small animal studies can be quickly examined in the human population and the neurophysiological basis of alterations observed in humans can be determined in animal models. Caution is necessary, however, particularly in with regards to maintaining animal physiology and accounting for the effects of anesthesia. In addition, the same considerations that hold for rs-fMRI as a biomarker in humans (Castellanos et al., 2013) remain for rodent studies. Validity, reliability, sensitivity, and specificity are key indicators of potential utility in both species.

The development of rs-fMRI techniques for rodents has opened the door for the creation of new knowledge in the fields of neuroscience, psychiatry, and neurology. In the basic sciences, we expect that multimodal studies in rodents will continue to disentangle the processes that contribute to the BOLD signal fluctuations and functional connectivity, allowing us to selectively sensitize our analysis to processes of particular interest for a given condition. In the clinical arena, we are already seeing the development of connectomic biomarkers for rodent models of brain disorders. As the field of pathoconnectomics matures, we can only expect the combination of functional connectivity mapping with genetic models may have a synergistic interaction that springboards our understanding of brain disorders to a new level. We are also enthused about the recent combination of optogenetics and MRI, where rs-fMRI can allow a network-level, whole brain evaluation of a very specific, local stimulation. Similar work may allow us to obtain new information about the mechanisms behind changes associated with invasive and non-invasive brain stimulation techniques, facilitating their application in human studies.

## Funding

This work was supported by NIH R01 NS078095 and NIH R21 NS072810.

### Conflict of interest statement

The authors declare that the research was conducted in the absence of any commercial or financial relationships that could be construed as a potential conflict of interest.
